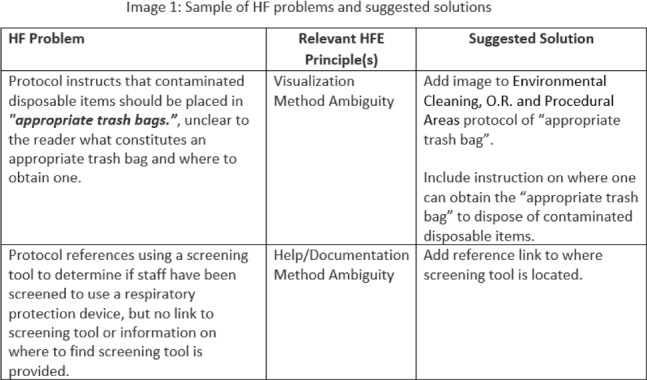# Development of a human factors-based tool for evaluating and improving infection prevention and control protocols

**DOI:** 10.1017/ash.2022.173

**Published:** 2022-05-16

**Authors:** Emma MacIntyre, Shawna Perry, Patience Osei, Raymond Terhorst, Ayse Gurses

## Abstract

**Background:** Infection prevention and control (IPC) protocols and guidelines are important quality management tools for educating care professionals and standardizing care processes. However, most of the actual care (ie, work as done) differ from protocol recommendations (ie, work as imagined). No tool or set of criteria has been established for how to develop human-centered IPC protocols. The goal of this research was to develop a standardized human-factors analysis method to provide healthcare organizations with a tangible framework to improve protocol usefulness and usability. **Methods:** The proposed analysis method combines principles from human-factors engineering (ie, usability heuristics, systems ambiguity framework) and instructional design. Relevant literature was analyzed by experts in human factors and clinical experts to develop a tool with criteria such as visualization and method ambiguity. Overall, 5 IPC-related protocols from a large academic hospital were selected from an electronic database and were evaluated using the proposed criteria. **Results:** During application of the analysis method, 70 human-factors–related problems were identified across 5 IPC protocols (eg, heater cooler cleaning), including violation of design heuristics and the presence of ambiguity. Frequently violated human-factors design principles included appearance and/or visibility (ie, visual display of content), visualization (ie, providing illustrative examples), and method ambiguity (ie, lack of clarity on how to complete a task). Figure 1 provides a sample of the human-factors problems identified and suggested solutions. Only minor modifications (ie, clarification of criteria definitions) were needed on the final tool. **Conclusions:** The human-factors–based tool developed in this study can be used both to develop new IPC protocols and to evaluate and improve existing protocols.

**Funding:** The CDC funded this work. This material is based upon work supported by the Naval Sea Systems Command under Contract No. N00024-13-D-6400, Task Order NH076. Any opinions, findings and conclusions or recommendations expressed in this material are those of the author(s) and do not necessarily reflect the views of the Naval Sea Systems Command (NAVSEA) or the US CDC.

**Disclosures:** None

**Figure:**